# mRNA vaccines for cancer immunotherapy

**DOI:** 10.3389/fimmu.2022.1029069

**Published:** 2022-12-14

**Authors:** Yashavantha L. Vishweshwaraiah, Nikolay V. Dokholyan

**Affiliations:** ^1^Department of Pharmacology, Penn State College of Medicine, Hershey, PA, United States; ^2^Department of Biochemistry and Molecular Biology, Penn State College of Medicine, Hershey, PA, United States; ^3^Department of Chemistry, Pennsylvania State University, University Park, PA, United States; ^4^Department of Biomedical Engineering, Pennsylvania State University, University Park, PA, United States

**Keywords:** mRNA, cancer vaccine, immunotherapy, lipid nanoparticles, nucleic acid, optimization, tumor antigen

## Abstract

Immunotherapy has emerged as a breakthrough strategy in cancer treatment. mRNA vaccines are an attractive and powerful immunotherapeutic platform against cancer because of their high potency, specificity, versatility, rapid and large-scale development capability, low-cost manufacturing potential, and safety. Recent technological advances in mRNA vaccine design and delivery have accelerated mRNA cancer vaccines’ development and clinical application. In this review, we present various cancer vaccine platforms with a focus on nucleic acid vaccines. We discuss rational design and optimization strategies for mRNA cancer vaccine development. We highlight the platforms available for delivery of the mRNA vaccines with a focus on lipid nanoparticles (LNPs) based delivery systems. Finally, we discuss the limitations of mRNA cancer vaccines and future challenges.

## 1 Introduction

With almost 10 million deaths per year, cancer remains one of the leading causes of death worldwide (1). Finding effective means to fight cancer has been one of the main goals of researchers worldwide for decades and still presents us with enormous challenges. In recent years, immunotherapy has been emerging as a major cancer treatment strategy (2–4). Immunotherapy is a therapeutic approach that dynamically modulates the immune system to recognize and destroy cancer cells. Various immunotherapy approaches are being developed to improve clinical outcomes in cancer patients. The development of cancer vaccines is a promising immunotherapy strategy to induce tumor antigens (TAs) specific and long-lasting immune responses. The artificial triggering of an immune response against TAs forms the basis for vaccines against cancers (1, 5).

Cancer vaccines target TAs to elicit both cellular and humoral immune responses which suppress tumor growth and eradicate the tumor (6). TAs can be classified into tumor-associated antigens and tumor-specific antigens. Tumor-associated antigens are nonmutated proteins that are overexpressed or aberrantly expressed in cancer cells (7). Tumor-associated antigens can be differentiation antigens, products of silent genes, universal tumor antigens, and oncoviral antigens. Clinical trials of cancer vaccines targeting tumor-associated antigens have had limited success (8). In some cases, tumor-associated antigens are expressed in normal cells, increasing the risk of vaccine-induced autoimmune toxicity. Tumor-specific antigens are specifically displayed by the tumor cells and are generally not displayed by the normal cells (9). Neoantigens are unique, tumor-specific antigens, resulting from the genetic instability of cancer cells (10). Neoantigens have a higher affinity for major histocompatibility complex (MHC) and potent immunogenicity. They are specifically expressed by tumor cells and elicit a tumor-specific T-cell response with limited “off-target” toxicity. Hence, neoantigens have become the main target for cancer vaccines in recent years (11).

Cancer vaccination strategies are of two types: preventive or prophylactic strategy and therapeutic strategy (12, 13). The preventive strategy aims to induce immune memory by administering vaccines to healthy individuals to prevent morbidity due to virus-associated cancers. There are currently only two prophylactic vaccines that are approved by the FDA to prevent malignancies caused by hepatitis B virus and human papillomavirus (11, 14, 15). However, not all cancers can be avoided by prophylactic vaccinations, as not all cancers are caused by viruses. To date, no preventive vaccine against non-viral cancers has been approved for use in humans. The therapeutic strategy aims to treat the disease by boosting or reactivating the patient’s own immune system. Two therapeutic vaccines are currently approved in cancer immunotherapy, namely the Bacillus Calmette-Guérin (BCG) vaccine for bladder cancer and a dendritic cell-based vaccine (Sipuleucel-T) for castration-resistant prostate cancer (11). In addition to the approved cancer vaccines, several other cancer vaccines are either in development or in the preclinical and clinical research phase (16). A complete list of cancer vaccines in clinical trials is available at clinicaltrials.gov.

Despite considerable research into cancer vaccine development, the clinical use of cancer vaccines has been hampered due to the diversity of tumor antigens, systemic toxicity, and low immunogenicity of tumor antigens. In recent years, in-depth studies of immunological mechanisms and the development of various new vaccine platforms have greatly advanced vaccine research. The rapid development and success of RNA-based vaccines against SARS-CoV-2 in response to the COVID-19 pandemic have brought cancer vaccines back into focus.

In this review, we discuss cancer vaccine approaches with a focus on nucleic acid vaccines, compare DNA and mRNA cancer vaccines, and finally discuss on the approaches for designing and optimizing mRNA-based cancer vaccines, delivery formats for mRNA vaccines, and future prospects.

## 2 Cancer vaccine platform types

**Figure 1:**
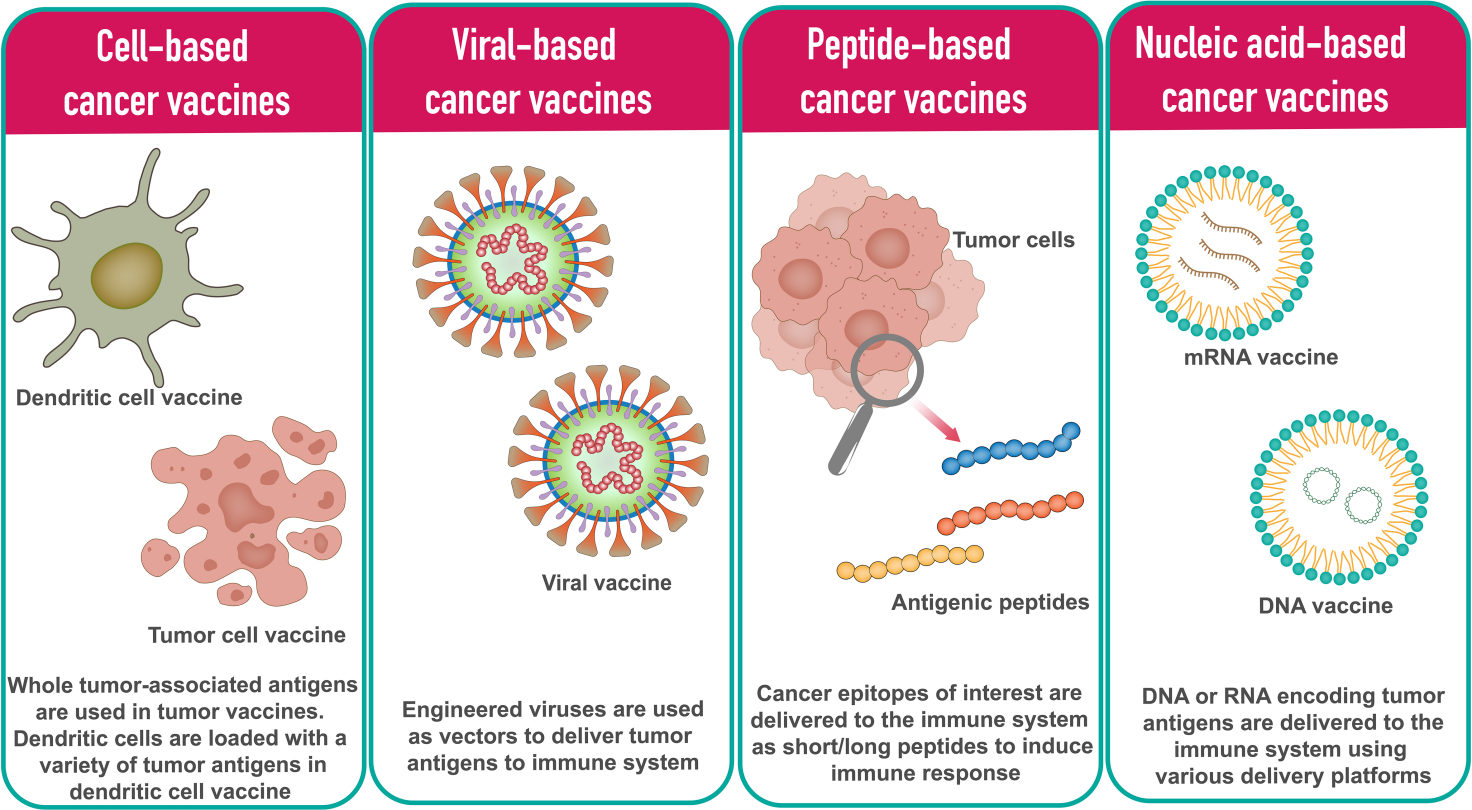
Different types of cancer vaccine platforms.

In general, cancer vaccine platforms are classified into cell-based vaccines, peptide-based vaccines, viral-based vaccines, and nucleic acid-based vaccines (**Figure 1**).

### 2.1 Cell-based cancer vaccines

The tumor cell vaccine approach is a simple and straightforward method in which allogenic or autologous patient-derived tumor cells are used to produce cellular vaccines (17, 18). To enhance the immune response against whole tumor cells, tumor cell lines can be genetically modified by introducing cytokines, chemokines, and co-stimulatory molecule-encoding genes or by silencing immunosuppressive genes. The limitation of this method is that it is sometimes difficult to obtain a sufficient number of cells to induce effective immune response (19).

Dendritic cells (DCs) are highly specialized antigen presenting cells (APCs) that activate naive T cells and are used in the development of cell-based cancer vaccines (20). In DC based vaccine development approach, DCs are loaded with a variety of tumor antigens in the form of DNA, RNA, tumor lysates, tumor-derived proteins, or peptides. Based on the DCs subpopulation, various types of DC vaccines have been developed in recent years. The main types of DCs used in DC vaccines include monocyte-derived DCs (Mo-DCs) and leukemia-derived DCs (DCleu) (20). Since it is possible to culture DCs in adequate numbers, DC cancer vaccines have been tested in phase I, II and III clinical trials (21).

### 2.2 Peptide-based cancer vaccines

Peptide-based cancer vaccines consist of highly immunogenic tumor-specific peptide antigens to elicit the desired immune response. Using synthetic peptides, peptide vaccination approaches are being used to develop personalized cancer vaccines. Upon administration, peptides antigenic peptides are taken up by APCs and presented in complex with the HLA molecules on the cell surface. T cells recognize the surface antigens, leading to cancer-specific immune responses. The peptide-based vaccine approach has several advantages over other types of vaccines, particularly in terms of safety and ease of manufacturing (22). HBV and HPV vaccines for liver and cervical cancers are two examples of peptide-based vaccines (23).

### 2.3 Viral-based cancer vaccines

Many viruses are inherently immunogenic, and their genetic content can be manipulated to include sequences encoding TAs. Several viruses have been used as platforms for cancer vaccines. The most common viral vaccine vectors are from adenoviruses, poxviruses, and alphaviruses (24, 25). Most viral vectors are either replication-defective or attenuated versions. A major advantage of virus-based vaccines is that the immune system responds efficiently to viruses, with both innate and adaptive mechanisms working together in the induction of strong and durable immune responses (26). The downside is that the antiviral immune response can neutralize the vector, limiting further repeat immunizations.

Oncolytic virus vaccines represent a novel and exciting approach. Oncolytic viruses identify, infect, and kill tumor cells and promote anti-tumor responses. After infection with the oncolytic virus, tumor cells produce reactive oxygen species (ROS) and cytokines that stimulate immune cells, followed by oncolysis (27–29). T-VEC, a first-generation recombinant herpes simplex virus product, is one such oncolytic virus vaccine (30). Besides herpes simplex virus, adenovirus is another commonly used oncolytic virus due to its ease of handling and a broad spectrum of host cell tropism (11).

### 2.4 Nucleic acid-based cancer vaccines

Nucleic acid vaccines are vaccines that contain antigens encoded by either DNA or RNA. The nucleic acid vaccine is a promising and attractive vaccine platform because it allows multiple antigens to be easily administered with one immunization and its ability to induce strong MHC I mediated CD8+ T cell responses (31). Compared to traditional vaccines, nucleic acid vaccines have demonstrated advantages such as safety, specificity for inducing the immune response for the antigen of interest, induction of both humoral and cellular immune responses, relatively low production cost, and ease of manufacturing (32).

DNA cancer vaccines consist of engineered DNAs that code for one or more TAs. DNA vaccines cross the cell membrane of APCs to the cytoplasm and move to the nucleus to start transcription. The resulting mRNAs translocate to the cytoplasm where they are translated into specific TAs by the host machinery. The resulting antigens are then presented to APC to stimulate an immune response (33). Poor immunogenicity of DNA vaccines compared to other vaccine platforms and long-term expression have drawn attention to RNA vaccines (34). Several DNA cancer vaccines have undergone preclinical and clinical trials over the past decade. The DNA vaccine has been extensively studied in cervical cancer. VGX-3100, a DNA vaccine against HPV-16/HPV-18 E6 and E7 oncogenes, has shown promising results in patients with premalignant high-grade cervical intraepithelial neoplasia (35). This vaccine is currently being evaluated in two Phase III clinical trials for safety and efficacy. GX-188E is another cervical cancer DNA vaccine that fuses multiple epitopes (36). GX-188E has the ability to target and activate dendritic cells. Promising results were obtained in a phase II study of GX-188E in cervical cancer (36). Recently, a preclinical study using a synthetic DNA multi-neoantigen vaccine demonstrated a therapeutic antitumor response by inducing a predominant CD8+ T cell response in mouse tumor models (37). In addition, DNA cancer vaccines have demonstrated safety and tolerability in early clinical trials for the treatment of multiple prostate and breast cancers (38, 39).

Like DNA vaccines, mRNA vaccines deliver genetic information encoding TAs in the form of mRNAs. mRNA vaccines do not need to reach the nucleus as they are translated in the cytoplasm (40). The overall immunogenicity of mRNA vaccines is slightly better than that achieved with DNA vaccines. Transient expression of mRNA-encoded antigen allows for more controlled antigen exposure and reduces long-term antigen exposure risk. The disadvantage of the RNA vaccine is that RNA is more easily degraded than DNA (41). However, there are various modifications that can increase stability. Due to challenges related to stability, cost of personalized manufacturing of patient-specific vaccines, and delivery, advances in clinical development of mRNA vaccines have been slow. The COVID-19 pandemic led to the successful development and deployment of multiple mRNA vaccines, confirming the mRNA platform’s remarkable versatility, safety, and promising immunogenicity on a global scale (42).

Several mRNA cancer vaccines are in different phases of development. Immunostimulant mRNA vaccine TriMix, encoding CD70, CD40L, and a constitutively active form of TLR4 produced vigorous CD8+ T cell responses in patients with stage III or IV melanoma, showing favorable tumor response rates in phase II clinical trial (43). Another immunostimulant mRNA vaccine, mRNA-252, which encodes human OX40L, IL-23, and IL-36, was developed by Moderna for the treatment of lymphoma and is currently in a clinical trial (NCT03739931). BNT111 mRNA vaccine that encodes four TAAs (NY-ESO-1, MAGE-A3, tyrosinase, and TPTE) has been effective in the treatment of melanoma patients (44). BioNTech and Moderna’s personalized mRNA vaccines have shown promising anti-tumor effects in clinical trials. Currently, there are two personalized mRNA cancer vaccines, Moderna vaccine mRNA-4157 (encodes up to 34 neoantigens) and BioNTech vaccine BNT122 (encodes up to 20 neoantigens), in phase II clinical trials (45). A phase II clinical trial with BNT122 for the treatment of colorectal cancer is currently underway (NCT04486378).

For this review, we focus only on mRNA-based vaccines.

## 3 Rational design and optimization of mRNA cancer vaccines

**Figure 2:**
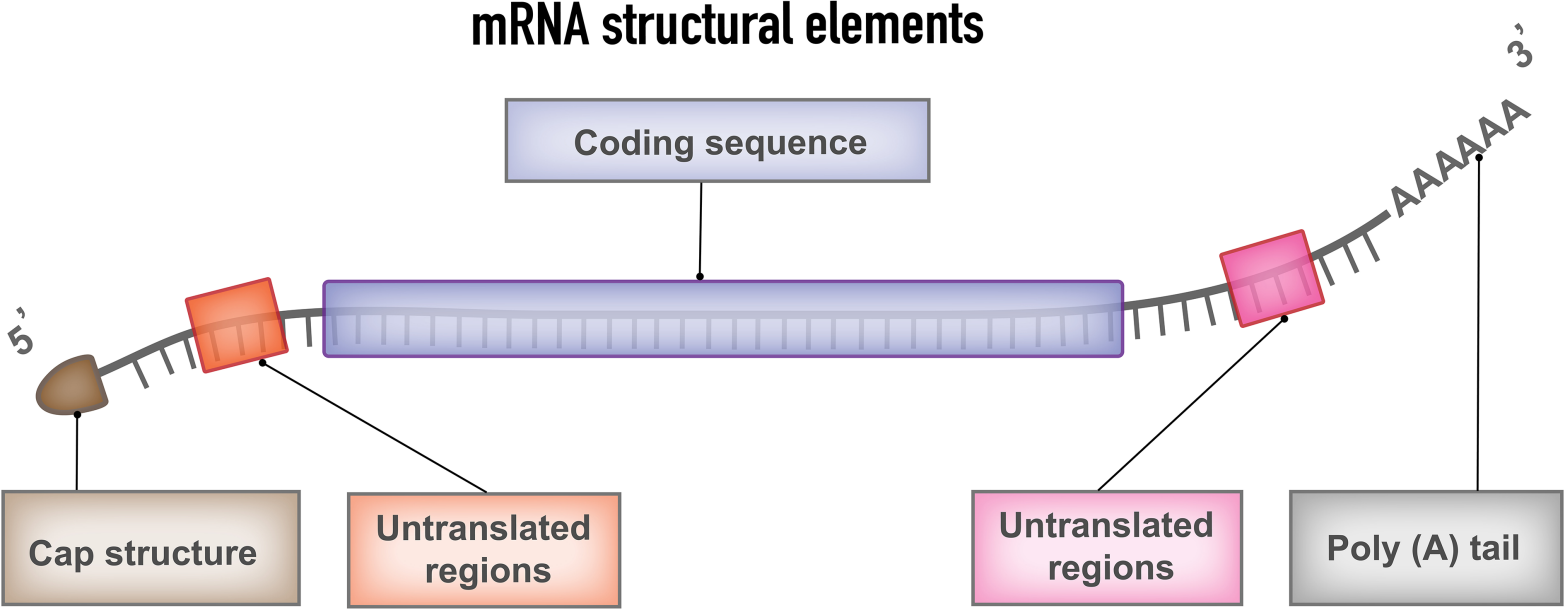
mRNA structural elements. Structural elements of mRNA vaccine include coding sequence, flanked by 5′and 3′ untranslated regions (UTRs), 5′ cap structure and 3′poly (A) tail.

The typical mRNA consists of a cap flanked by 5′- untranslated regions (UTR), 3′-UTRs, an open reading frame (ORF) encoding cancer antigens in mRNA cancer vaccines, and a poly(A) tail (**Figure 2**). These components of mRNA can be modified to increase stability, translational efficiency, and immunostimulatory properties. The design and optimization approaches include design and optimization of the coding region, design, and optimization of the noncoding region, and design and optimization of delivery formats.

### 3.1 Design and optimization of the coding sequence

It is known that codon composition affects translation efficiency. Substituting the rare codons with regular synonymous codons that contain many similar tRNAs in the cytosol accelerates translation and increases yield (46).

However, rare codon optimization for nucleic acid therapies may have potentially serious consequences that should be evaluated (47). Another form of sequence optimization is the enrichment of the GC content. GC enriched sequences are translated at rates 100-fold higher than low GC sequences (48). mRNA can be optimized by incorporating chemically modified nucleosides, which are known to decrease immunogenicity and significantly improve translational efficiency. Nucleotide modifications such as 5-methylcytidine (m5C), 1-methylpseudouridine and pseudouridine (ψ) are generally preferred modifications (49, 50).

### 3.2 Design and optimization of the noncoding region

The 5′ and 3′ UTR elements adjacent to the coding sequence are critical considerations in optimal vaccine design as they have a significant impact on mRNA stability, ribosome recognition, and translation (51). Optimizing 5′- and 3′-UTR elements greatly increases the efficiency and half-life of mRNA. The 5′-UTR sequence can be optimized by avoiding the presence of start codons in the 5′-UTR that disrupt ORF translation, by avoiding the presence of highly stable secondary structures that affect ribosome recruitment and codon recognition, by using shorter 5′-UTRs that are ideal for mRNA translation (52, 53). By introducing the 3′-UTRs of *α-*and β-globin mRNAs, translation and stability of mRNA may be enhanced (54).

The 5′-cap structure is essential for effective mRNA protein synthesis. The 5-cap regulates pre-mRNA splicing and nuclear export acts as a protective structure protecting RNA from exonuclease cleavage and initiates mRNA translation. 5′ capping can be achieved by using a vaccinia virus capping enzyme or by incorporation of synthetic cap or anti-reverse cap analogs during or after the transcription process (52, 55).

The poly(A) tail stabilizes the mRNA and promotes protein translation. The appropriate length of the poly(A) tail is crucial for the regulation of mRNA translation and stability (56). The length of the poly(A) tail is directly proportional to the translational efficacy. The poly(A) tail improves the stability of mRNA by slowing down the degradation of RNA by RNA exonucleases (45). There are two ways to add a poly(A) tail to *in vitro* transcribed (IVT) mRNA *i.e.* (i) extending the IVT mRNA after transcription by using recombinant poly(A) polymerase (ii) including poly(A) tail encoding DNA template from which IVT mRNA is transcribed. mRNA transcribed from a DNA template yields transcripts with a defined poly(A) tail length, whereas the enzymatic polyadenylation process yields mRNA transcripts with variable length poly(A) tails. In addition, deadenylation by poly(A)-specific nucleases can be inhibited by the incorporation of modified nucleotides into the poly(A) tail (52).

### 3.3 Delivery format optimization

After generating the IVT mRNA transcript, the next step is to administer the RNA vaccine, which should eventually reach the cytoplasm of the target cells. Because of the negatively charged structure of naked RNA and the large molecular size, mRNA is prone to degradation by nucleases and cannot cross the cell membrane. To overcome this obstacle, several mRNA vaccine delivery strategies have been employed, which can be broadly classified into two basic approaches *i.e*. (i) *ex vivo* loading of mRNA into DCs, (ii) direct injection of mRNA with or without a carrier.

#### 3.3.1 *Ex vivo* loading of mRNA into DCs

DCs are the most potent antigen-presenting cells in the immune system. When DCs are used as a vaccination platform, DCs are transfected with mRNA encoding a tumor antigen of interest and then delivered to the host to elicit an immune response against the antigen (57–59). DCs can be transfected with either TAAs mRNA or total tumor RNA (60, 61); both methods have their advantages and disadvantages. DCs can internalize naked mRNA through a variety of endocytic pathways, but *ex vivo* transfection is commonly enhanced by applying electroporation to achieve high transfection efficiency without the need for a carrier molecule (57). Once DCs are loaded with mRNA *ex vivo*, they are reinfused into the recipient of the autologous vaccine to elicit the immune response. Loading of DCs with additional mRNAs, such as mRNAs encoding costimulatory molecules CD83, tumor necrosis factor receptor superfamily member 4 (TNFRSF4), and 4-1BB ligand (4-1BBL), has been shown to result in a substantial increase in the immunostimulatory activities of DCs (59). Most *ex vivo* loaded DC vaccines elicit a predominantly cell-mediated immune response. *Ex vivo* DC loading allows precise control of transfection efficiency and cellular target. The main disadvantage of this approach is that it is an expensive and labor-intensive vaccination approach (49). An example of this approach is a phase I trial evaluating autologous Langerhans-type dendritic cells with xenogeneic TRP-2 mRNA (62).

#### 3.3.2 Direct injection of mRNA with or without a carrier

Direct injection of mRNA is a comparatively faster and less expensive approach. Recent advances in the direct injection approach have made a lot of progress in precise and efficient cell-type specific delivery of mRNA vaccines.

Naked mRNA has been used successfully for *in vivo* immunizations. Naked mRNA vaccines are formulated in buffer only and without a carrier. In this approach, native mRNA vaccines are injected directly. After administration, naked mRNAs can induce antigen-specific antibodies and T-cell immune responses (61). The limitation of the naked mRNA vaccine platform is the short extracellular half-life of naked mRNA due to rapid degradation caused by ubiquitous RNAases (63). Viral vector-based technologies have been used to deliver nucleic acid vaccines into cells, but their application is limited by pre-existing or vaccine-induced anti-vector immunity, which can reduce vaccine efficacy (64). To overcome some of these limitations, physical methods such as the gene gun method, electroporation, virus-like particles produced in yeast, synthetic delivery vehicles such as liposomes and lipoplexes, and cationic polymers have been developed for IVT mRNA to protect it from RNAase degradation, enhance cellular uptake and improve vaccine delivery (65–69).

**Figure 3:**
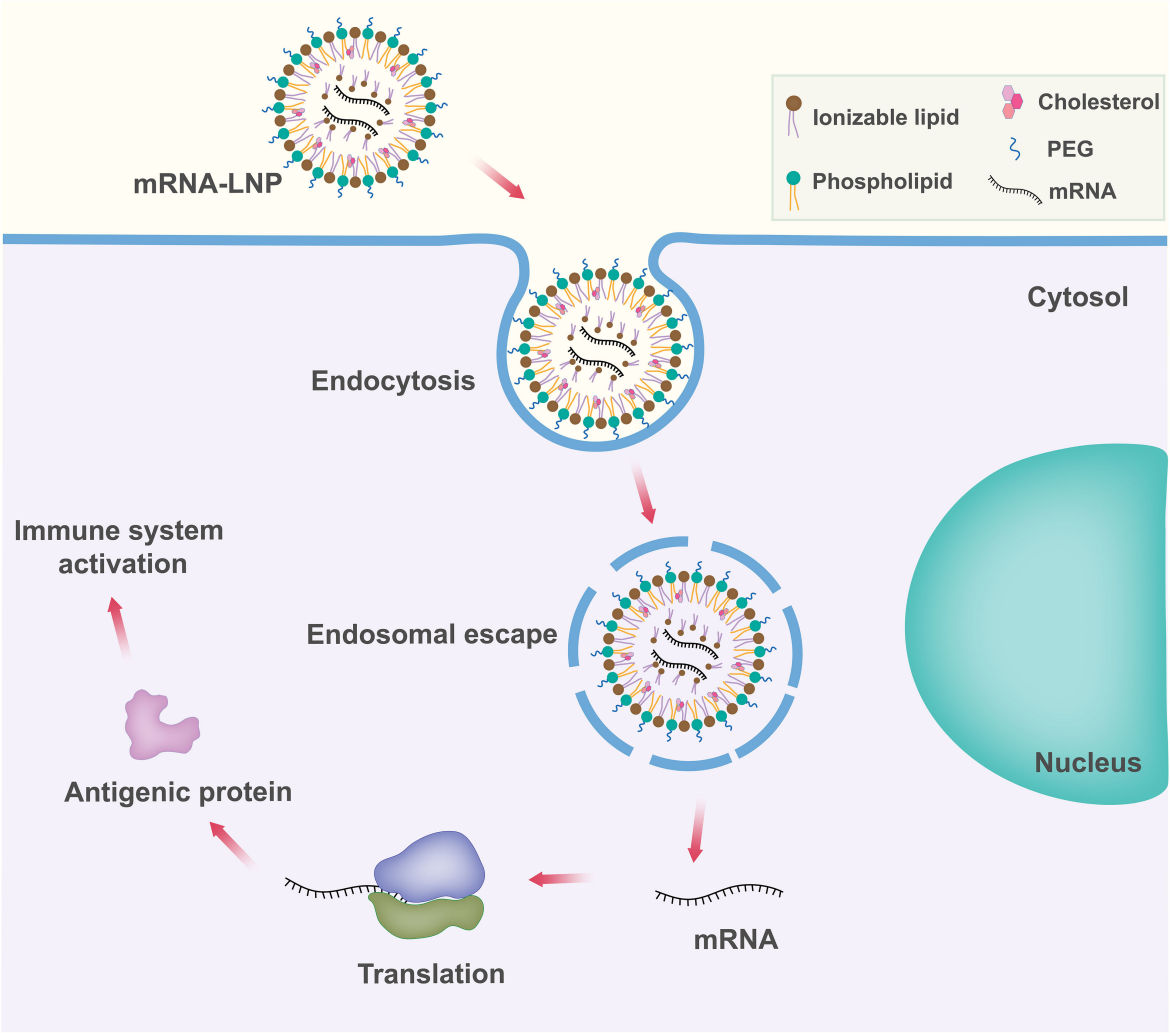
Schematic representation of lipid nanoparticle (LNP) based mRNA delivery. Components of the LNP are shown in the upper right box.

Among the various delivery vehicles, LNPs have emerged as one of the advanced and widely used mRNA delivery platforms due to the success of the mRNA-LNP vaccines against SARS-CoV-2 (**Figure 3**) (70). Lipid nanoparticles are nanosized lipid formulations designed to protect mRNA payloads from degradation and allow for their efficient delivery to target cells. These lipid-based nanocarriers can efficiently deliver mRNA intracellularly by fusing with the lipid bilayer of early endosomes, thereby transporting the mRNA into the cytosol. LNPs are typically ~100 nm size carriers and consist of four components: ionizable lipids to form complexation with mRNA and allow the endosomal release of mRNA to the cytoplasm; lipid-linked polyethylene glycol (PEG) to increase the half-life of formulations; cholesterol to stabilize the structure of LNP; and phospholipids to support the lipid bilayer structure (71, 72).

*Ionizable lipid.* Ionizable lipid is the most important component of LNP as it determines LNP potency. Ionizable lipid generally differentiates different mRNA-LNPs. Ionizable lipids consist of a hydrophilic head group, hydrocarbon chains to enhance self-assembly, and linkers to connect the head groups to the hydrocarbon chains. Ionizable lipids are essential for mRNA complexation. Ionizable lipids are unionized within the LNPs, and they complex with mRNA to form electrostatically stable lipoplex. Ionizable lipids remain neutral in the systemic circulation pH (pH~7.4), but become protonated at early endosomal pH (pH~6.5) and facilitate endosomal membrane fusion followed by cytosolic release (73–75). Ionizable lipids lack a substantial positive charge at physiological pH, resulting in improved pharmacokinetics (76). This property increases the half-life in the bloodstream, allowing for better accumulation in target tissues such as solid tumors. Some ionizable lipids are known to induce inflammation and cell toxicity by activating toll-like receptors (TLR) pathways (77).

*Polyethylene glycol (PEG)-lipid*. Polyethylene glycol lipids generally comprise <2.5% of the total formulation in LNP. PEG-lipid structure consists of a hydrophilic PEG-polymer, which is conjugated with a hydrophobic lipid anchor. They are found at the surface of LNPs with the lipid domain hidden down in the particle and the PEG domain protruding from the surface. PEG lipids play an important role in balancing circulation time and cellular uptake (71). They are also important for the proper determination of particle size during manufacture (78). PEG-lipid helps to inhibit particle aggregation and in turn improves storage stability (79). Balancing the PEG lipids is important because they are known to prevent the transport of RNA into cells at high concentrations. The development of anti-PEG antibodies has raised concerns about possible allergic reactions to LNPs (80).

*Phospholipids and cholesterol.* Phospholipids and cholesterol contribute to the structural integrity and phase transition behavior of the LNPs. Cholesterol and phospholipid components of LNPs are unlikely to elicit significant innate immune recognition and inflammatory responses as they are naturally present in mammalian cell membranes (81).

The main advantage of mRNA-LNP vaccines is the modularity and versatility of the platform. LNPs components and their ratios, targeting moieties, and overall lipid-to-mRNA ratios can be tailored and optimized for different targets and applications. LNPs have lower immunogenicity, can deliver larger cargoes, and offer opportunities for rapid and large-scale manufacture. However, more studies should be done on the risks of mRNA-LNP technology. As with most drugs, side effects with mRNA-LNP vaccines often increase with dose. For example, for the mRNA-1273 vaccine, 100 μg of the dose showed good efficacy and minimal side effects, and 250 μg of the vaccine caused severe side effects, while the BNT162b2 vaccine at 30 μg showed better efficacy and minimal side effects (82, 83). Anaphylactic reactions and inflammatory reactions have been observed with some COVID-19 mRNA-LNP vaccines, even at the recommended doses (84–86). In addition, there is a residual risk of toxic side effects associated with the complexing agents and delivery compounds. Long-term immunological changes affecting adaptive immune responses have been reported (87). These data necessitate future studies to optimize the delivery system of mRNA vaccines.

Apart from the platform, the route of administration is also important to the effectiveness of the mRNA vaccines. Intramuscular and intradermal injections are the most commonly used routes of injection because these routes of injection provide the highest level of immunity and the longest duration of effect (71). Intravenous administration involves liver first-pass metabolism and is less convenient, so it is less preferred (71). The systemic route is only preferred in select cases.

## 4 Concluding remarks

mRNA cancer vaccines are a powerful and versatile form of immunotherapy. mRNA cancer vaccines are able to encode and express TAA, TSA, and their associated cytokines, and these vaccines can induce both humoral and cellular immunity. Appropriate selection of antigens is the basis for the development of mRNA cancer vaccines. mRNA cancer vaccines have several advantages, such as rapid and large-scale production, flexibility, versatility, relatively low production costs, no oncogenic potential, well-tolerated, and the ability to elicit a robust protective immune response. Importantly, mRNA vaccines do not carry the risk of integrating into the host genome, making them a promising therapeutic modality. The viability of mRNA vaccines to fight cancer has been demonstrated by numerous preclinical and clinical studies. Various mRNA cancer vaccines are currently being developed for a variety of cancer treatments. These studies have been extensively reviewed (45, 51, 88, 89).

Personalized mRNA vaccines open a new direction for precision cancer therapy. Personalized mRNA cancer vaccines coding for specific cancer antigens can be produced by utilizing next-generation sequencing (NGS) technology. Various computational approaches can be used to predict neoantigens and their presentation by human leukocyte antigen (HLA). Previously, we demonstrated such an application of computational approaches in epitope prediction and rational vaccine design (42, 90–94). With the increasing number of studies and clinical trials of personalized cancer vaccines, the possibility of developing mRNA vaccines against different types of cancer is mounting. Despite the promise of mRNA cancer therapy, much more research is needed to develop stable mRNA and safe advanced delivery systems. Further development of personalized vaccines and clinical trials for different tumors are required.

mRNA-based vaccines have gained more and more popularity for the development of novel immunotherapies. However, the instability and *in vivo* delivery of mRNA cancer vaccine have impaired its clinical application. Although progress has been made over the past decades to overcome these limitations, challenges still exist on the development of mRNA cancer vaccines. Another major challenge is the targeted delivery of mRNA to specific tissues and cell types. In addition, future studies could focus on combining mRNA cancer vaccines with other immunotherapies to improve clinical outcomes and cancer treatment.

In summary, given the technological revolution in the field of mRNA vaccines, we can soon expect a leap in cancer immunotherapy and successful clinical translation of mRNA cancer vaccines.

## Author contributions

All authors listed have made a substantial, direct, and intellectual contribution to the work and approved it for publication.
